# Probabilistic Assessment of High-Throughput Wireless Sensor Networks

**DOI:** 10.3390/s16060792

**Published:** 2016-05-31

**Authors:** Robin E. Kim, Kirill Mechitov, Sung-Han Sim, Billie F. Spencer, Junho Song

**Affiliations:** 1 Fire Research Center, Korea Institute of Civil Engineering and Building Technology, Gyeonggi-do 18544, Korea; 2 Department of Computer Science, University of Illinois at Urbana-Champaign, Champaign, IL 61801, USA; mechitov@illinois.edu; 3 School of Urban and Environmental Engineering, Ulsan National Institute of Science and Technology (UNIST), Ulsan 44919, Korea; ssim@unist.ac.kr; 4 Department of Civil and Environmental Engineering, University of Illinois at Urbana-Champaign, Champaign, IL 61801, USA; bfs@illinois.edu; 5 Department of Civil and Environmental Engineering, Seoul National University, Seoul 08826, Korea; junhosong@snu.ac.kr

**Keywords:** wireless sensor networks, network communication reliability, probabilistic assessment, high-throughput data transfer, structural health monitoring

## Abstract

Structural health monitoring (SHM) using wireless smart sensors (WSS) has the potential to provide rich information on the state of a structure. However, because of their distributed nature, maintaining highly robust and reliable networks can be challenging. Assessing WSS network communication quality before and after finalizing a deployment is critical to achieve a successful WSS network for SHM purposes. Early studies on WSS network reliability mostly used temporal signal indicators, composed of a smaller number of packets, to assess the network reliability. However, because the WSS networks for SHM purpose often require high data throughput, *i.e.*, a larger number of packets are delivered within the communication, such an approach is not sufficient. Instead, in this study, a model that can assess, probabilistically, the long-term performance of the network is proposed. The proposed model is based on readily-available measured data sets that represent communication quality during high-throughput data transfer. Then, an empirical limit-state function is determined, which is further used to estimate the probability of network communication failure. Monte Carlo simulation is adopted in this paper and applied to a small and a full-bridge wireless networks. By performing the proposed analysis in complex sensor networks, an optimized sensor topology can be achieved.

## 1. Introduction

Due to the local nature of damage, structural health monitoring (SHM) often requires a densely-distributed sensor topology. However, traditional wired sensors are challenging in establishing an appropriate sensor network; cabling in the system requires high labor and increases the total cost [1]. Wireless smart sensors (WSS), on the other hand, offer the promise of low-cost and ease of installation. WSS’ capability of on-board processing of raw data allows overcoming challenges of data inundation arising from high data throughput in a WSS networks. Due to such features, numerous researchers have deployed short-term and long-term monitoring systems on structures using commercially available and academic prototype sensors. Summaries and examples of WSS development and full-scale monitoring can be found in [2,3,4,5,6,7,8,9,10,11,12,13,14,15,16,17,18]. Despite these successful deployments, full-scale implementations are still limited. 

One of the critical issues hindering broader use of WSS for SHM is securing a reliable network for high-throughput data. For low- *vs.* high-throughput applications, the difference is between environment monitoring applications and data-intensive applications, such as SHM [19]. For the former, the data may be composed of several packets and, thus, can be easily streamed as it is generated due to the small amount of information that needs to be transmitted. However, in the latter case, in which the data is used for system identification or damage detection of a structure, communication requirements are completely different. For example, a network composed of more than hundreds of wireless sensors could command collecting 20 min of tri-axial acceleration time history at 50 Hz sampling frequency. In such a data intensive application, data is being generated much faster than it can be sent. Moreover, failures occurring during the data transfer typically result in loss of data, significantly degrading the accuracy of monitoring that can be carried out [20]. Thus, ensuring reliable network communication for high-throughput data applications is important.

Various recent researchers have investigated the communication performance of low-power WSS, mostly focusing on low-throughput applications. A part of the comprehensive studies on the characterization of network communication can be found from the following literature [21,22,23,24,25,26,27]. As an example, Huang *et al.* [28] reported on a probabilistic approach to characterize the network communication failure as a function of increased communication range. Jang *et al.* [26] identified the potential source of signal attenuation, using the T-R separation distance, by collecting data from the radio signal strength indicator (RSSI) and link-quality indicator (LQI), packet delivery rate (PDR), construction materials, and the thickness. Although most of the aforementioned literature have successfully identified key factors affecting wireless communication, most of the applications were for a temporal link (low-throughput data), rather than for a long term communication, *i.e.*, high-throughput data [21]. For example, Bocca *et al.* [25] measured signal strength during wireless communication, but interpretation of the measurements over a larger amount of data was not drawn. Thus, to date, practical methodologies for assessing reliability of WSS networks for high-throughput SHM WSS network has been lacking. 

In this paper, we propose an approach to assess reliability of a WSS deployed for high-throughput WSS networks. Following a review of the existing WSS platforms (with focuses on MICA2 and Imote2 platform) and the requirements for SHM applications, a framework for assessing the reliability of WSS networks is presented. One of the most common tools for assessing the reliability of a system, *i.e.*, Monte Carlo simulation (MCS), is employed along with collected data. Examples using lab-scale and full-scale WSS networks are provided to demonstrate the sensitivity of the model and practical use of the proposed approach. Since the wireless sensors rely on radio communication, the network can be intrinsically unreliable. However, by using the proposed approach, a quantitative assessment of the wireless network can be obtained. In addition, an optimized sensor topology can be achieved that will be able to deliver robust and reliable performance in complex sensor networks.

## 2. Problem Description

To better understand the need for reliability assessment, this reviews existing wireless platforms widely used for WSS networks for SHM purposes. Subsequently, the unpredictable communication behavior of WSS nodes is described using test results with a simple application.

### 2.1. WSS Platforms for SHM Systems

Careful selection of the fundamental hardware for WSS nodes is required for the appropriate design of WSS networks for SHM. Low energy consumption when operating embedded software is the most critical, because sensors run on battery power. Significant transmission range, user-selectable radio channels, fast data rate, and low-power demand are also desirable features. To implement novel SHM features requiring that response data be processed and stored, WSS should possess a fast microcontroller, as well as sufficient RAM (random access memory) and ROM (read-only memory). A number of academic prototypes and commercially available platforms have been developed to meet such requirements and adopted by researchers for SHM [2,29].

Many existing WSS originated from DARPA’s (Defense Advanced Research Projects Agency) “Smart Dust” project in the late 1990s [30,31]. One of these WSS nodes was the Mica family of low-power motes developed by researchers at the University of California at Berkeley [32]. For example, the Mica2 platform ran on only two AA batteries for months with a low-duty cycle [33,34]. However, Spencer *et al.* [29] reported that because the platform had limited storage capacities, those platforms are inappropriate for SHM purposes. Aside from this class of WSS, Intel introduced a data-intensive platform, the Imote2 [35], which could meet the demands of SHM applications. The following paragraph discusses hardware and software of the Imote2 in more detail. 

When first introduced, the Imote2 aimed to provide powerful computational capabilities. The Imote2 features a PXA27x chip, Intel’s low-power X-scale processor. The processor is a multi-chip module with 256 KB SRAM, 32 KB of ROM, and 32 MB of Flash memory [36]. For radio communication, the Imote2 uses a Chipcon CC2420 2.4 GHz IEEE 802.15.4 RF transmitter. The radio chip aims to provide a high data rate and robust communication with its transmission power output ranges from −25 dBm to 0 dBm [36,37,38]. As in many WSS platforms, the Imote2 employs TinyOS as its operating system [39]. Within the software application, the default packet for TinyOS consists of four components: a preamble, header, data, and footer, and is a fixed size of 28 bytes. 

Spencer *et al.* [40] reported the potential suitability of the Imote2 for SHM purposes among other platforms; subsequent research developed extensive SHM hardware and software for the Imote2 platform. Under the ISHMP (Illinois Structural Health Monitoring Project), a series of high-fidelity sensor boards and a library of middleware services, including time synchronization and reliable communication, were developed [1,19,20,41]. However, as will be discussed in the next section, regardless of how an application aims to provide reliable service, the application may fail due to the unpredictable nature of WSS communication links.

### 2.2. Illustration of Unpredictable WSS Performance

**Figure 1:**
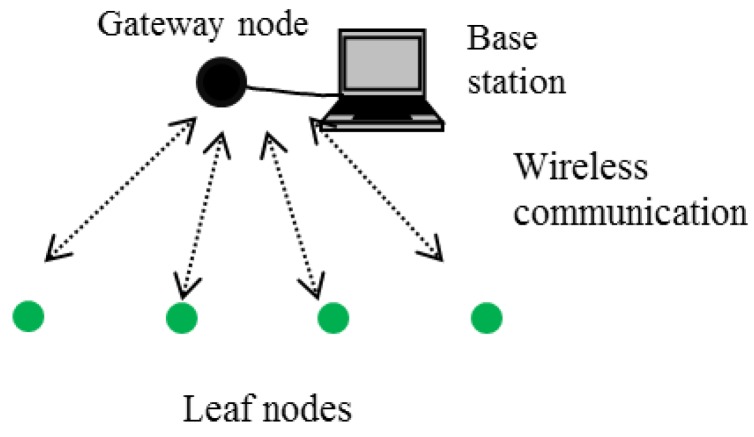
Illustration of a wireless network.

This section illustrates the unpredictable performances of a WSS platform, Imote2s, even under identical communication circumstances. Applications in the ISHMP Services Toolsuite aim to provide robust performance. Figure 1 shows an example of a WSS network. When a command is sent from a central base station to leaf nodes in its network, the individual leaf nodes resend a packet to the base station once the application is successfully completed. If the message does not arrive within a certain amount of time, the base station acknowledges that the application has failed at the leaf node. Such a failure may be caused by (i) the failure of the node while performing the tasks; (ii) the failure of communication such that the commands from the base station have not arrived; or (iii) the failure of communication such that the acknowledgement message from the node is not received. Reset (an Arial font herein indicates the name of an application) is one of the simplest applications among those services. A message from the base station node only contains a reset command. The acknowledge message will be immediately sent back once the command is received. Due to its simplicity, the application failure will provide a good indicator of communication failure during sending/receiving the messages.

**Figure 2:**
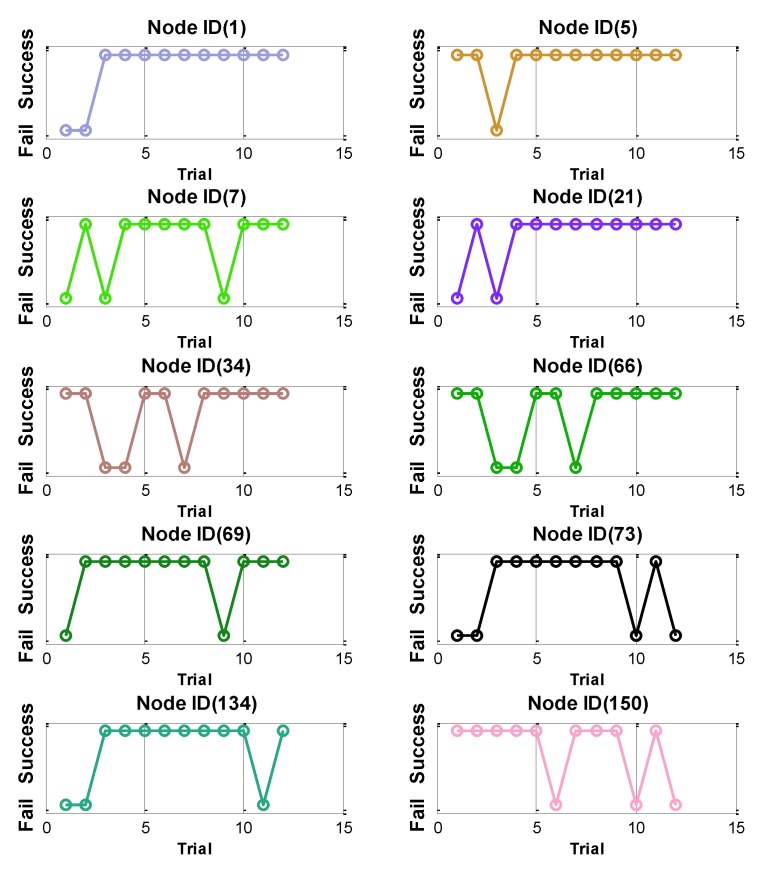
Application test under identical communication circumstances.

Ten Imote2s were prepared in an outdoor environment, in which all leaf nodes are located about 100 m from the base station node. Stable DC power was provided to the nodes from an 80-Watt power supply. Thus, the communication environment and the power source were identical. The test performed 12 trials, in which the base station broadcasted the reset command to ten leaf nodes. Figure 2 summarizes the returned packets from each leaf node acknowledging the completion of Reset. Although the application is simple and the communication circumstance was identical, each leaf node showed different performance. The number of failures and the trials at which a sensor failed varied among sensors. Node ID (5) had only one failure, while Node ID (73) failed four times. The test results indicate that the performance of an application may vary with each nodes and also with other unknown factors. This type of communication uncertainty is always present in the wireless communication network. To use such failure-prone WSS in a SHM application requiring high data throughput, appropriate models for these failures, as well as a means to analyze their impact on the overall network, must be developed.

## 3. Measures of High-Throughput Communication Quality

This section investigates high-throughput communication performance of a wireless platform to understand the relationships between the sensor performance and commonly-adopted link quality indicators. In this paper, the Imote2 platforms are used, but the approach can be applied to any type of a wireless sensor platform.

Various researchers have proposed statistical methods to characterize the quality of the wireless links. Those studies used indices that can provide instantaneous measures of link instability and radio signal strength, such as the radio signal strength indicator (RSSI) and link-quality indicator (LQI). RSSI is the measure of power in the received radio signals and LQI estimates how easily the signals can be modulated in the presence of noise in the channel [42]. Numerous researchers have related the radio communication quality to packet reception rates (PRR) by measuring RSSI and LQI [43,44]. Although previous studies have found some correlation between those indices, such relationships provide only instantaneous measures of link characteristics. Therefore, those methods are not directly applicable to WSS networks for SHM applications. 

An application, TestRadio, that outputs measured RSSI, LQI values at the sender and receiver nodes were used to identify the extended relationships between those indices and the long-term data transfer performance of the wireless link. A built-in radio chip in the Imote2 can provide digital RSSI and LQI values. The dynamic range of RSSI is from −100 dB to 0 dB and LQI ranges from 0 to 255 [38]. The gateway node sent 1000 packets to the leaf node. Upon receiving the packets, the leaf node returned the packets to the gateway node, with information about RSSI and LQI of each packet. Returned RSSI (rrssi, a Courier New font herein indicates the parameter shown in the TestRadio application) and returned LQI (rlqi) represent communication qualities at the leaf nodes. Then the gateway calculates PRR (r%) using the ratio between received and sent packets, which indicates the measure of data loss. Thus, PRR can be a measure of reliability and goodness of the links. In this example, the gateway node aimed to transmit 1000 packets and received 1000 packets back, thus, r% is 100%. Output of TestRadio indicates high-throughput data transfer performance of the nodes because the application provides cumulative r% and averaged rrssi and rlqi over a number of packets that were communicated between the gateway node and the leaf node.

**Figure 3:**
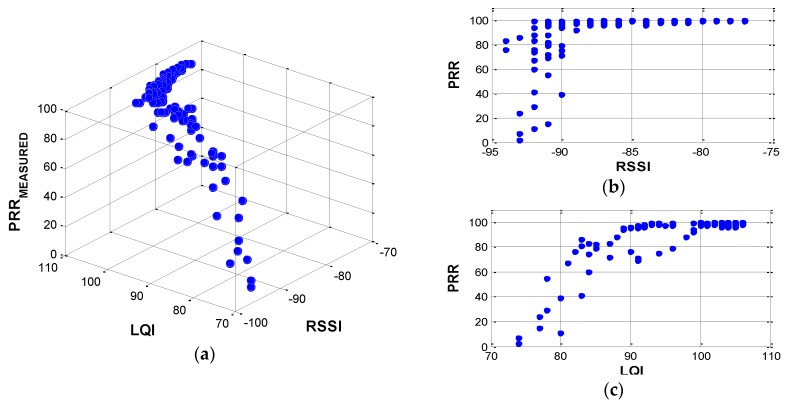
Relationships among PRR, RSSI, and LQI: (**a**) RSSI-LQI-PRR; (**b**) RSSI-PRR; and (**c**) LQI-PRR.

TestRadio was performed extensively using three leaf nodes to measure averaged rrssi (noted as RSSI hereafter), rlqi (also noted as LQI) and r% (averaged PRR). The test setup is summarized in Table 1. In this test, hardware related bias was reduced by using three different Imote2s. Figure 3 shows three-dimensional and two-dimensional relationships among PRR-LQI-RSSI. The overturned ‘L-shape’ relationships, which PRR decreases sharply as both RSSI and LQI value decreases are observed. 

**Figure 4:**
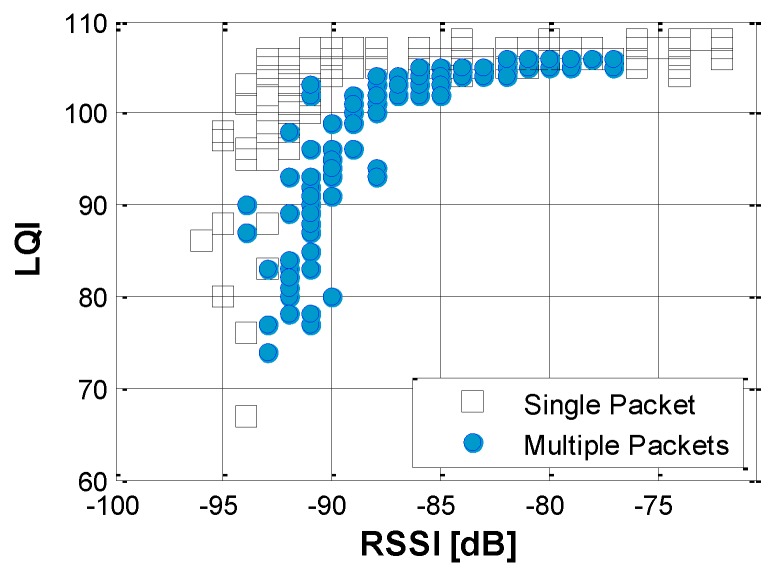
Relationships of RSSI and LQI in comparison between single and multiple packets being used in the communication.

The sharper edge in PRR-RSSI (Figure 3b) indicates that RSSI itself does not accurately predict PRR due to high fluctuations in the domain RSSI < −90 and due to low sensitivity with higher RSSI (*i.e.*, RSSI > −90). Note that the relationship is constructed based on averaged RSSI and LQI among 1000 packets to represent the large data throughput, for SHM purposes. The results using multiple packets show the better correlation between RSSI and LQI when compared with relationship constructed using a single packet (see Figure 4); note that the gray region in the single packet case is sharper than that found from the multiple packet case.

**Figure 5:**
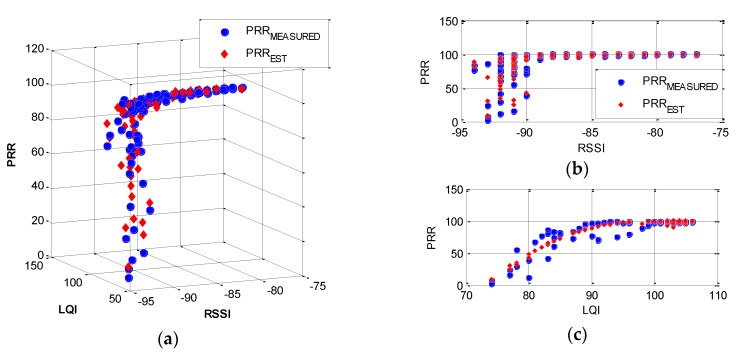
Regression of PRR: (**a**) RSSI-PRR; (**b**) LQI-PRR; and (**c**) RSSI-LQI-PRR relationship.

Estimated PRR, *PRR_EST_*, is expressed as a function of RSSI and LQI, based on the relationships observed in the measured data. An appropriate model should be computationally efficient, represent PRR well, and have a physically reasonable boundary. For example, using a second-order polynomial model:

(1) $$PRR_{EST}\left(x_{RSSI},x_{LQI}\right)=a+b(x_{RSSI})+c(x_{LQI})+d(x_{RSSI}{}^{2})+e(x_{RSSI})(x_{LQI})+f(x_{LQI}{}^{2})$$

where *a*, *b*, *c*, *d*, *e*, and *f* are constants obtained from nonlinear regression of the measured PRR on the measured RSSI, *x_RSSI_*, and LQI, *x_LQI_*. The goodness of the model is examined by R^2^. As shown in Figure 5, the regression model captures trends of the measured PRR over the measurement range of RSSI and LQI reasonably well with R^2^ = 0.87. However, because of the randomness in *x_RSSI_* and *x_LQI_*, an empirical equation in Equation (1) alone cannot represent their relationship with PRR accurately. Thus, using Equation (1), a stochastic approach will be adopted in assessing the network communication quality in the next section.

## 4. Framework for Probabilistic Assessment of Network Performance

This section defines a framework for the probabilistic assessment of WSS networks using the relationship identified in the earlier section. The limit state function is defined first and then the uncertainty is added to the model. 

### 4.1. Limit State Function

A failure state of a leaf node in SHM applications is defined as an event that a leaf node fails to send measured signals to a gateway node due to an excessive amount of packet loss occurring during the transmission. Knowing packet loss before sending data, however, is challenging. Instead, the PRR can be estimated, *PRR_EST_*, using empirical relationships shown in Equation (1), and compared with a threshold PRR, *PRR_LIMIT_*. Equation (2) shows such a state; when *PRR_EST_* is lower than *PRR_LIMIT_*, the communication environment is considered harsh and a leaf node is likely to give up completing data transmission to the base station:

(2) $$g\left(x\right)=PRR_{EST}\left(x_{RSSI},x_{LQI}\right)-PRR_{LIMIT}+\varepsilon$$

In the equation, the vector, *x*, basic variables of the problem (*i.e.*, *x_RSSI_*, and *x_LQI_*), and the term ε, represent the epistemic error of the model. One can adopt the central limit theorem to compensate for such a model error. The distribution model of ε is assumed to follow the normal distribution with zero mean and standard deviation, σ, determined during regression analysis ε(*x_RSSI_*, *x_LQI_*)~N(0, σ). A linear regression method is used to find an unbiased estimation of the conditional variance of *ε* for given values of RSSI and LQI [45]. Then, a constant for *PRR_LIMIT_* should be determined, depending on application requirements. For example, a high number should be used when the application aims for near real-time data acquisition and each packet is irreplaceable. On the other hand, if an application can retransmit the same packet to assure the packet has been received, a relatively low constant can be used. Then, a set, the collection of all outcomes in the sample domain, will be determined by either a failure set when *g*(*x*) is lower than 0, a limit state when *g*(*x*) = 0, or a safe set when *g*(*x*) > 0.

A number of different methods can be adopted to solve the limit-state function with random variables. Such methods include Monte Carlo simulation (MCS), and the first and second order reliability methods, and asymptotic techniques [46]. Among various methods, one of the most popular reasons for using MCS is because it is a powerful tool to estimate the failure probability; MCS allows direct consideration of any type of probability distribution, and is easy to implement at the desired precision [47]. Thus, in this paper, MCS will be applied in assessing the network reliability. The distribution of the random variables, RSSI and LQI, and brief introductions on MCS will be described in the next section.

### 4.2. Modeling the Uncertainties in the Network

This section determines the distribution types of random variables in Equation (2) using the measured RSSI and LQI in the test described in Table 1. In selecting distribution models, the maximum likelihood estimation method was adopted with the following criteria:

1. The shape of the selected probability density function (PDF) of a random variable should represent that of the histogram well.
2. The boundary of each distribution must not violate physical limitations of random variables.

**Figure 6:**
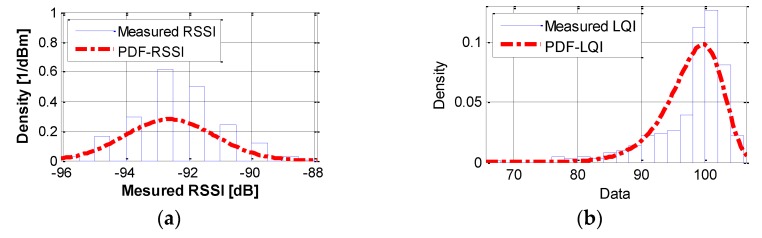
Histogram and suggested PDFs: (**a**) RSSI; and (**b**) LQI.

For example, from the measured datasets, the PDF of RSSI can be a normal distribution (see Figure 6a) with mean value (μ*_RSSI_*) of −92.60 dB and standard deviation (σ*_RSSI_*) of 1.43. For PDF of LQI, the gamma distribution (see Figure 6b) can be used with the following parameters: μ*_LQI_* = 97.50, σ*_LQI_* = 36.24, *a_LQI_* = 262.335, and *b_LQI_* = 8.6 × 10^−4^. Note that *a_LQI_* and *b_LQI_* refer to the shape and scale parameters of the gamma distribution, respectively. The histogram of RSSI has discontinuities, as can be seen in Figure 6, because RSSI is a quantized integer value. The mean and variances of RSSI and LQI are susceptible to environmental changes, such as the distance and nearby material types. However, distribution types are less likely to change because hardware-related biases are considered by using three different Imote2s. Thus, the usage of the application will collect smaller amounts of data to find the parameter fitting for the distribution types. This approach enhances in-place applicability.

Finally, probability of a failure is defined as follows:

(3) $$P_{f}=\int_{x\in \Omega_{E}(x)}f(x)dx$$

where *P_f_* denotes the probability of failure, *f*(*x*) is a standard joint normal PDF, and Ω*_E_*(*x*) is the failure domain determined by limit-state functions.

In the case when the example to be applied show not quite small expected *P_f_*, *i.e.*, the system is not very reliable and *P_f_* > 10^−4^. The MCS method can be preferred [48]. In the approach, MCS introduces an indicator, *I*, whose value equals one if *g*(*x*) is less than zero, or if the sample space lies in the failure domain and *I* = 0 when *g*(*x*) is larger than zero [49]. 

A large number of samples will increase the accuracy of *P_f_*, but computational demands can be high. To determine the precision of *P_f_* and an efficient number of samples, the coefficient of variation (c.o.v., δ*_Pf_*) is usually realized.

In summary, the reliability model to assess the high-throughput wireless communication quality has been determined; a limit state function using the measured variables and estimated PRR are defined. Distribution types for random variables are also chosen using the measured data. Here, the tests contain a wide range of LQI and RSSI, reduced hardware-related bias, and achieved the following: (i) a deterministic distribution shapes of the random variables and PRR, which can reduce the number of trials for assessing the network periodically; and (ii) a reliability model that is robust from unknown temporal factors that may influence the communication quality, such as noise or jitter. However, constants used in Equation (1) may be sensitive to the long-term changes in the environment; *i.e.*, distance from the gateway to leaf nodes, radio blockage, *etc.* Therefore, the proposed approach will require *a priori* tests with a smaller number of datasets for the following reasons: (i) determine the characteristics of two random variables (RSSI and LQI); and (ii) curve fit the predetermined equation to obtain constants in Equation (1). The following section will demonstrate the applicability of the proposed method to assess reliability of wireless networks.

## 5. Case Studies

Among various influences affecting the communication quality of the WSS network, the communication range is one of the most easily controllable factors. Thus, this section first evaluates the performance of the developed model in an outdoor small-scale test to validate that the model is sensitive to capture the degradation of the network reliability as a function of communication range. Then, the proposed approach has been applied for assessing a more complex full-scale WSS network, where unknown factors may contribute to degradation of the reliability of the network communication.

### 5.1. Case 1: Small-Scale WSS Network

**Figure 7:**
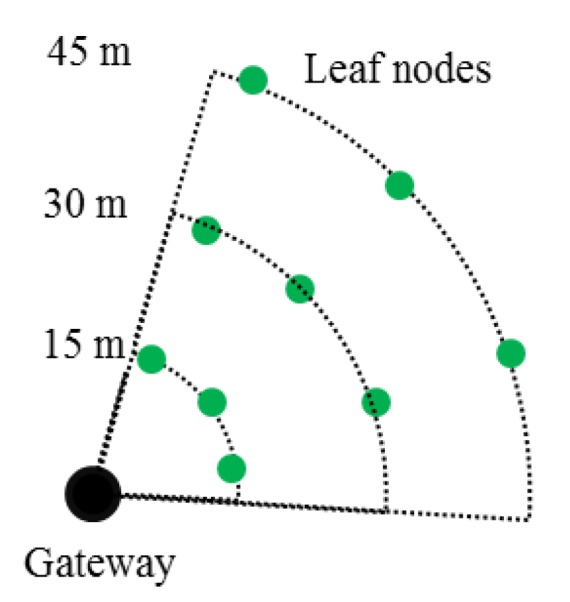
Small-scale test topology.

A temporal outdoor WSS network was prepared to assess the performance of the model developed in the Section 4. Due to the simplicity of the test setup in a small-scale test, environments that may affect the communication, such as interference from external signals and/or physical objects, *etc.* have been controlled to provide that the approach is sensitive in capturing the probability of failures as a function of distance from leaf nodes to a gateway node. In total, nine leaf nodes were prepared at three different locations; *i.e.*, at each distance, three leaf nodes were located to mitigate hardware-related bias (Figure 7). Three locations were measured from the base station, which were 15, 30, and 45 m. Leaf nodes were lifted about 1 m above from the ground to remove the signal blockage [50]. A Li-ion polymer battery provided a stable power of 3.7 V and 10 Ah to all sensors. Within the network, all leaf nodes and the gateway node had the identical external antenna with a peak gain of 2.2 dBi [51].

A set of radio tests determined the parameters for the distributions of two random variables and curve-fitted the limit-state function in Equation (2). The test averaged RSSI and LQI values over 100 packets and performed 100 times. From heuristic relationships between packet loss rate and the successful collection of a long data set from a leaf node to a gateway, PRRLIMIT was set to 85%. 

**Figure 8:**
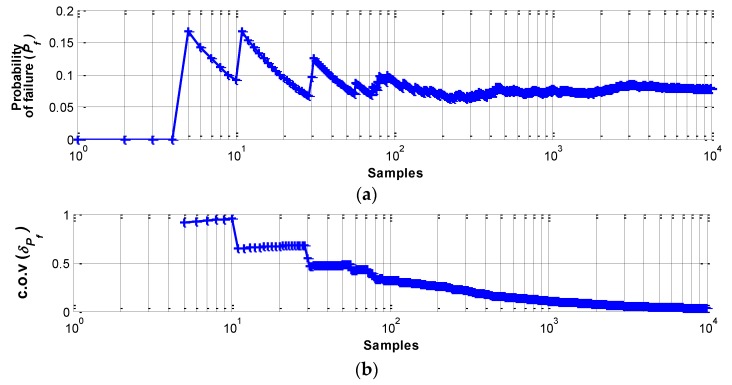
MCS results; (**a**) Probability of failure (*P_f_*); and (**b**) c.o.v. ($\delta_{P_{f}}$).

Then, MCS has been applied on the radio test data to check the applicability of the simulation in assessing the network reliability. During the simulation, δ*_Pf_* validated the simulation size. Convergence of δ*_Pf_* to a relatively small value indicates that a sufficient number of samples has been generated. For example, Figure 8 shows the converging *P_f_* and δ*_Pf_* for test results obtained from the 15 m WSS. Approximately 10^4^ samples gives confidence for the *P_E_* and the size is used for simulating 30 and 45 m test sets.

Table 2 summarizes *P_f_* obtained from MCS (104 samples). As the distance from the base station increases, *P_f_* gradually increases, validating the sensitivity of the proposed model as a function of distance. The fast convergence rate in the example (as can be seen from Figure 8), allows using large sample sizes and confirms that MCS analysis can be a powerful and faithful tool for obtaining accurate results. 

### 5.2. Case 2: Full-Scale WSS Network on the Jindo Bridge

#### 5.2.1. Network Description

**Figure 9:**
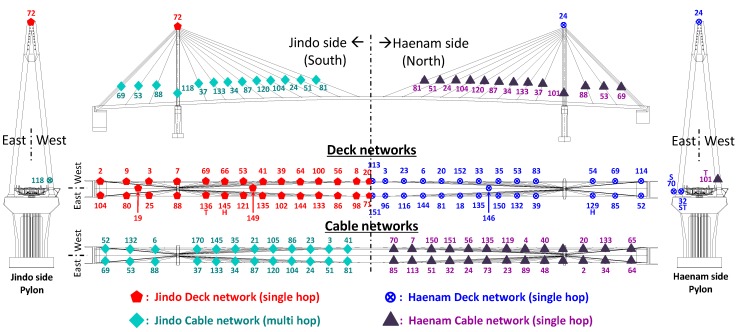
Jindo Bridge WSS networks layout.

**Figure 10:**
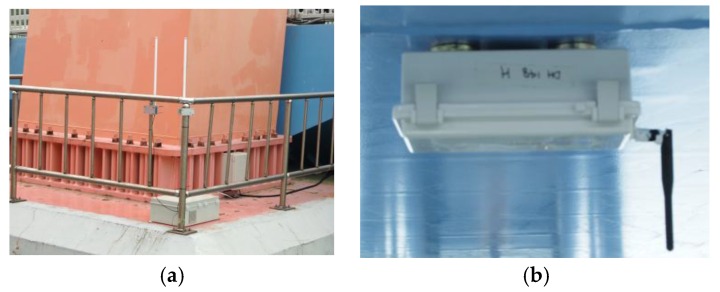
Illustration of the (**a**) Base station and (**b**) Wireless node.

The world’s largest full-scale WSS network was deployed on the second Jindo Bridge, Korea using the Imote2 platform and the ISHMP Services Toolsuite. The primary purpose of the network is to demonstrate the capability of measuring high-throughput data for long-term monitoring of the structure using WSS [1,10]. The bridge is a steel cable stayed bridge, which connects the Jindo Island and Korean Peninsula (see Figure 9). The fact that the bridge is made of steel, which blocks the passage of the radio signals, and that the bridge is often subject to high humidity due to frequent fog and harsh winds, which weakens the signal, interference in the radio communication was expected to be high. Thus, some efforts were made in the design of WSS networks for the Jindo Bridge to enhance the network communication. The entire network was decomposed into four subnets with two central base station PCs to reduce the number of nodes in each subnet and the distance between the base station and the respective nodes. Radio transmission channels were selected that were not in use by other users. The maximum communication distance from a central server to a WSS was no more than 170 m (note that the Imote2 can cover up to 300 m distance at its ideal condition). However, because the results from the small-scale network indicated that the failure of transferring large amounts of data may be up to 20% with sensors being placed over 30 m, two types of antennas were used to enhance the communication quality. Each base station sensor, which all sensor nodes in each sub-network transfer data packets to, had an 8 dBi antenna (see Figure 10a, PM-0M07 8 dB Volcado, Daeheung, Seoul, Korea) whereas the other sensor nodes the other sensor nodes deployed on the bridge had 2 dBi (see Figure 10b, PM-DI02A, Daeheung, Seoul, Korea). Among four subnets, a subnet on the bridge deck near the Haenam side (shown in a box in the Figure 9) is selected in this paper as a benchmark in assessing the reliability of the network.

#### 5.2.2. Performance Evaluation

**Figure 11:**
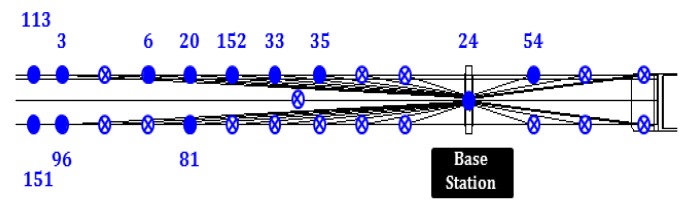
Sensor topology used for WSS networks reliability assessment.

The reliability model has been applied to the full-scale WSS network infrastructure deployed on the Jindo Bridge. Figure 11 shows the distribution of WSS in the selected subnet. The base station for this subnet was on the pylon pier at the Haenam side. As appears in Figure 10a, the base station has an antenna with higher gain than the leaf nodes. However, the developed model assesses the network using the communication quality indicators estimated at the leaf node side, which has a lower gain antenna.

Table 3 summarizes the results from MCS method at each WSS in the sub-network. With converging c.o.v., obtained *P_f_* values are very small regardless of the distance from the base station. The sub-network is highly robust and reliable. The result indicates that the extra antenna installed at the base station enhanced overall communication quality of the subnet. However, the *P_f_* at Node Id 3 is as high as 30.596%. This result illustrates the uncertainty among WSS nodes and that the understanding the reliability of the WSS network based on each sensor’s performance is important. Identifying such a failure-prone sensor can be improved by further actions. For example, the antenna on Node Id 3 can be replaced with a higher-gain antenna or be replaced to a nearby location to the base station to maintain a similar degree of PE over the sub-network.

At the same time, in a case when the entire network shows low *P_f_* the lower radio power on the leaf node can be selected to reduce the excessive power use. Additionally, the network design can be optimized to have subnets when covering larger civil infrastructure.

## 6. Conclusions

Low-cost and on-board computational capabilities of wireless smart sensors (WSS) have shown potential for wide applications for monitoring civil infrastructure. However, due to the distributed and unreliable nature of WSS, maintaining highly robust and reliable networks is challenging. Especially, WSS for structural health monitoring (SHM) require large amounts of data throughput making the realization of such a network more challenging. Thus, quantitative assessment of WSS network communication quality before and after finalizing a deployment is critical to achieve a successful WSS network. This paper’s proposed model assessed the reliability of the WSS network using readily available indicators. Using measured indices from a WSS, one of the most popular methods, Monte Carlo Simulation (MCS) analysis, assessed the failure probability (*P_f_*) of the network reliability, which is defined as data transfer failure due to excessive packet loss. The proposed method first identified the distribution characteristics of the selected two random variables, radio signal strength indicator and link quality indicator, and related them to the packet reception rate. The reliability of the measurement was enhanced by averaging over a number of datasets and performing an extensive number of test sets. Then, the packet reception rate was estimated using two random variables and determined the failure state of the data transfer in the limit state function. The error in the regression model has been compensated following the central limit theorem. The proposed approach has been validated from two case studies; (1) a small-scale temporal WSS network; and (2) a full-scale network. In a small-scale test, an environment that can affect the communication quality, excluding the range, has been controlled. The results of the study provided the proposed method is sensitive in capturing the increase of communication range between leaf nodes and a gateway node. Then, MCS has been applied to assess the network reliability of the Jindo Bridge, Korea, at which the sensor locations were pre-defined. The strengthened radio power and extra antenna device on the base station realized a robust WSS network, showing low PE on a subset of the Jindo Bridge. The proposed approach showed an effective way to assess the reliability of WSS networks with high-throughput data transfer. The approach can be also used in designing and optimizing the sensor topology for a long-term WSS network for SHM purpose. For example, employing the proposed method in the design phase can determine the distances between sensors, radio power, the types of antenna and its orientation. A periodic assessment can localize a sensor with communication errors and provide a more robust network. For example, because communication quality is susceptible to humidity, which depends on the environment of the system, the network can be assessed to incorporate seasonal variations. Inspecting reliability of the WSS network using the proposed approach in a design phase and during operation will lead to a successful long-term SHM using WSS.

## Figures and Tables

**Table 1:** Radio test setup.

Setup	Number
Number of lead nodes	3
Distance from a gateway (m)	5
Packets sent in each trial	100
Number of trials	500

**Table 2:** MCS results.

Distance from the Base Station (m)	*P_f_* (%)
15	7.712
30	20.130
45	28.551

**Table 3:** MCS results.

*Node Id*	*c.o.v.*	*P_f_* [%]	*Node Id*	*c.o.v.*	*P_f_* [%]
**54**	0.086	0.135	**81**	0.048	0.436
**24**	0.018	2.956	**6**	0.060	0.285
**35**	0.046	0.458	**3**	0.005	30.596
**33**	0.044	0.478	**96**	0.035	0.009
**152**	0.040	0.653	**113**	0.051	0.075
**20**	0.041	0.569	**151**	0.033	0.009
